# Assessment of Genetic Diversity and Population Structure of Exotic Sugar Beet (*Beta vulgaris* L.) Varieties Using Three Molecular Markers

**DOI:** 10.3390/plants13212954

**Published:** 2024-10-22

**Authors:** Bowei Sun, Shengnan Li, Zhi Pi, Zedong Wu, Ronghua Wang

**Affiliations:** 1 Academy of Modern Agriculture and Ecological Environment, Heilongjiang University, Harbin 150080, China; chuibaodiqiu@163.com (B.S.); 2020046@hlju.edu.cn (S.L.); 2018060@hlju.edu.cn (Z.P.); 2 Shihezi Academy of Agricultural Sciences, Shihezi 832061, China

**Keywords:** sugar beet varieties, molecular markers, genetic diversity, population structure

## Abstract

Sugar beet (*Beta vulgaris* L.) is a biennial herb belonging to the Amaranthaceae family. It contributes to approximately 30% of the world’s total sucrose production and is an economically important crop. In this study, we analyzed the genetic diversity and population structure of 132 exotic sugar beet varieties using three molecular makers: four pairs of simple sequence repeat (SSR) primers, three pairs of insertion–deletion sequence (InDel) primers, and 20 cis-element amplification polymorphism (CEAP) primers. The results indicated that the number of alleles (Na) was 298, among which the number of effective alleles (Ne) was 182.426 (accounting for approximately 61.2%). The mean value of the genetic diversity index was 0.836. The polymorphic information content (PIC) was 0.639–0.907 (mean = 0.819), indicating a high level of polymorphism. These sugar beet varieties were classified into six clusters using the UPGMA method of cluster analysis. Population structure analysis revealed that the most ideal K value was 6. This indicated that the test materials could be divided into six categories, consistent with the clustering results. The clustering results indicated that most sugar beet varieties from the same breeding company clustered together, and the genetic distance between them was small, indicating that they may share the same male and/or female parent. Some varieties from different companies clustered together, indicating a narrow genetic base and potential exchange of germplasm resources between breeding companies. This study revealed the genetic differences among exotic sugar beet varieties and characteristics of the population structure. It provided a scientific basis for the identification of sugar beet varieties and markers-assisted breeding in China in the future.

## 1. Introduction

Sugar beet (*Beta vulgaris* L.), belonging to the Amaranthaceae family [1], is a cultivated species that originated from the European region. Sugar beet is one of the world’s most economically valuable sugar crops and an important source of cane sugar in temperate regions of the Northern Hemisphere. Sugar beet contributes to approximately 30% of the world’s total sugar production [2]. Sugar beet exhibits cold and alkali resistance, which contributes to significant economic benefits. As an important sugar crop in China, the annual sugar production from sugar beet accounts for approximately 14% of the total sugar production in the country [3]. The dominant production areas of sugar beet in China are mainly located in the north, northwest, and northeast and other areas north of 40° N latitude, with the total planting area and production accounting for more than 95% of the national total in the country [4]. Cultivated beets are usually divided into four categories according to their usage: sugar beets, leaf beets, edible beets, and forage beets [5]. Among them, sugar beet is the main cultivated variety in agricultural production. Sugar beet is a biennial herb [6] that grows in temperate climates. It can be used to produce not only edible sugar but also raw materials for the production of methanol, ethanol, and acetone [7]. Moreover, the byproducts after sugar production can be utilized to produce a variety of secondary goods. In addition, the stems, leaves, and hair and tail roots of beets can be used to extract betaine and to manufacture agricultural feed [8].

Beet is a cross-pollinated crop, exhibiting self-incompatibility and a high degree of heterogeneity within the population. Therefore, its original genetic integrity is susceptible to various factors [9]. In 1906, sugar beet was introduced to China, becoming the earliest source of sugar beet genes [10]. The genetic improvement of crops and related research depends on the quantity and quality of existing germplasm resources. In addition to quantity and quality, the in-depth study of germplasm resources directly affects the efficiency of their utilization and sustainable development of the modern seed industry [11]. However, the existing sugar beet germplasm resources in China are not complete or adequate; the kinship relationship among germplasm resources is relatively close, and the genetic base is narrow. This limits sugar beet breeding in China [12].

Currently, almost all sugar beet varieties used in China are exotic and are regarded as monoembryonic types. Therefore, it is of great significance to understand their genetic diversity and population structure. The results of relevant studies are favorable for the construction of fingerprints and guiding the selection and breeding of superior sugar beet varieties in China. With the development of science and technology, molecular marker technology has become more advantageous [13] than morphological marker [14], cellular marker [15], and biochemical marker [16] technologies. Various molecular marker techniques such as SNP [17], RFLP [18], RAPD [19], lSSR [20], insertion–deletion sequence (InDel) [21], and simple sequence repeats (SSR) [22] have been widely used in recent decades in the study of genetic variation in several crops. Among them, SSR or microsatellite DNA markers are the most widely used because they are simple and convenient to use, can help in determining the size of alleles via nondenaturing polyacrylamide gel electrophoresis, and provide a large amount of genetic information through the large number of alleles at each locus [23]. Therefore, they provide a basis for studying genetic relationships, population structure, and genetic diversity of crops. InDel markers are third-generation molecular markers based on whole-genome sequencing, which are length polymorphic variations generated by the insertion or deletion of a relatively short nucleotide sequence in a certain number of nucleotides at an allelic locus [24]. The number of bands amplified by InDel molecular marker technology is small and highly recognizable, which reduces the possibility of subsequent identification errors caused by excessive clutter of bands [21]. Peng et al. [25] analyzed the genetic diversity, population structure, and cluster analysis of 129 sugar beet germplasm resources to screen superior germplasms for breeding using the 27 simple sequence repeat (SSR) and 33 pairs of insertion–deletion (InDel) molecular markers. Liang et al. [26] assess the genetic variation and genetic structure of 111 sugar beet varieties by utilizing simple sequence repeat (SSR), restriction site amplified polymorphism (RSAP), direct amplification of minisatellite DNA by PCR (DAMD), and start codon targeted (SCoT) molecular markers. Patel et al. [27] used 14 pairs of SSR and 21 pairs of InDel primers to analyze the genetic diversity of 19 colored and white rice genotypes and reported the highest genetic diversity between genotypes Krishna Kamod (white pericarp) and IRST 1 (red pericarp) and the lowest genetic diversity between genotypes Lal Kada (red pericarp) and Krishna Kamod (white pericarp). DNA markers are widely distributed in the genome, independent of the environment, and could be recognized at any developmental period and in any tissue.

The newly developed cis-element amplified polymorphism (CEAP) marker in recent years is a novel promoter and gene-targeted molecular marker based on cis-elements that are highly conserved among species [28]. It contains eight cis-elements: AAAG, ACGTG, CCGA, ACTCAT, GGTCA, TATCC, TGAC, and GATAA, which are closely related to plant growth and development, signaling, and response to adversity. Through cloning and sequencing, it was verified that the CEAP marker could be amplified from the promoter region, promoter and gene region only at the base polymorphisms due to region, gene, and 3′ non-coding regions. Chen [29] used CEAP molecular markers to amplify eight mango germplasm resources via PCR and reported that CEAP primers could amplify clear and high polymorphic bands. In addition, CEAP markers could well amplify germplasm resources of rice, tomato, potato, winter melon, citrus, and longan, and the primers had good generalization for the species [29]. The use of CEAP markers for genetic diversity analysis, kinship identification, and marker-assisted breeding in plants is a novel, simple, and low-cost method, and CEAP markers are universal among different species.

In this study, we aimed to understand the genetic diversity and population structure of 132 varieties of exotic sugar beet using three different kinds of molecular markers, namely, SSR, InDel, and CEAP, with 27 pairs (bands) of core primers. This study provided support for the selection and breeding of sugar beet germplasm resources in the future and the foundation for fully exploring and utilizing the excellent sugar beet genetic resources and formulating new hybrid combinations.

## 2. Results

### 2.1. Analysis of Genetic Diversity

A total of 298 alleles (Na) were detected in 27 pairs (bands) (Table 1), and 5 (AAAG28) to 19 (D32) alleles were amplified from each pair (band) primers with an average of 11 alleles. The average effective allele (Ne) value was 6.757, and the highest allele effective number of primers was 11.035 (TGAC28). The Shannon information index (I) ranged from 1.3689 (AAAG28) to 2.549 (TCAC26) (average 2.044). The maximum and minimum values of the observed heterozygosity (Ho) were 1.000 (TGAC23) and 0.207 (ACGTG4), respectively. The maximum and minimum values of the expected heterozygosity (He) were 0.913 (TGAC28) and 0.688 (AAAG28), respectively. Nei’s expected heterozygosity ranged from 0.683 (AAAG28) to 0.909 (TGAC28). The polymorphism information content (PIC) of the primers ranged from 0.639 (AAAG28) to 0.907 (TGAC26) (average 0.819), and all PIC values were >0.5. Among them, the PIC values of primers TCAC26 and AAAG28 were the highest and lowest, respectively.

### 2.2. Analysis of Population Structure

**Figure 1:**
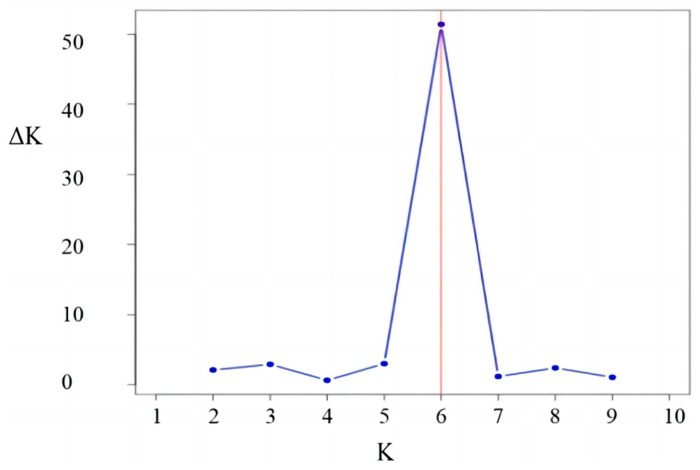
The magnitude of ∆K as a function of K. The corresponding ∆K value (K = 6) statistics determined the optimal structural allocation for 132 sugar beet varieties.

**Figure 2:**
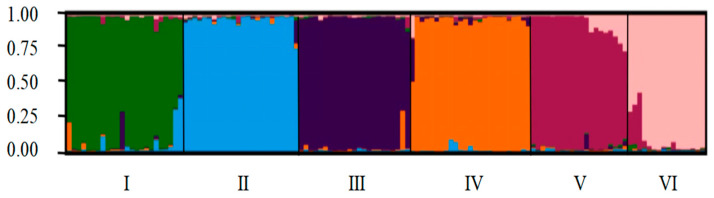
Population genetic structure of 132 sugar beet varieties at K = 6 based on four bands of SSR, three pairs of InDel, and 20 CEAP markers. At K = 6, the population was divided into six (I, II, III, IV, V, and VI) based on STRUCTURE analysis.

The maximum value of ∆K occurred at K = 6 (Figure 1). This indicated that the 132 sugar beet varieties could be divided into six different groups (Figure 2). The six different colors corresponded to six subgroups (I, II, III, IV, V, and VI), among which most sugar beet varieties in subgroup I were from Maribohilleshog ApS. The sugar beet varieties in subgroups II, III, IV, and V were mostly from Maribohilleshog APS and SES VanderHave, Maribohilleshog ApS, KWS SAAT SE, and SES VanderHave, respectively.

### 2.3. Genetic Distance and Cluster Analysis

In conformity with the outcomes derived from the PCR amplification, an extensive analysis of the samples of exotic sugar beet varieties was conducted utilizing the MEGA7 software application. Consequently, the genetic distance metrics for 132 sugar beet varieties were ascertained, as detailed in Table S1. The genetic distances among the 132 samples spanned a range of 0.065 to 0.287, with an average value of 0.207. Moreover, the genetic distances among the predominant sugar beet cultivars were observed to cluster within the range of 0.19 to 0.23, as documented in Table S1. Most sugar beet varieties from the same company exhibited a small genetic distance between each other and were clustered into a class. For example, sugar beet varieties 89 and 90 (both from Maribohilleshög ApS) exhibited a genetic distance of 0.081; the varieties 30 and 31 (both from KWS SAAT SE) exhibited a genetic distance of 0.106 (Table S1). However, in some cases, the genetic distance between sugar beet varieties from different companies was small. For example, the genetic distance between the varieties 103 and 107 (from SES VanderHave and BETASEED, respectively) was 0.065 (Table S1).

**Figure 3:**
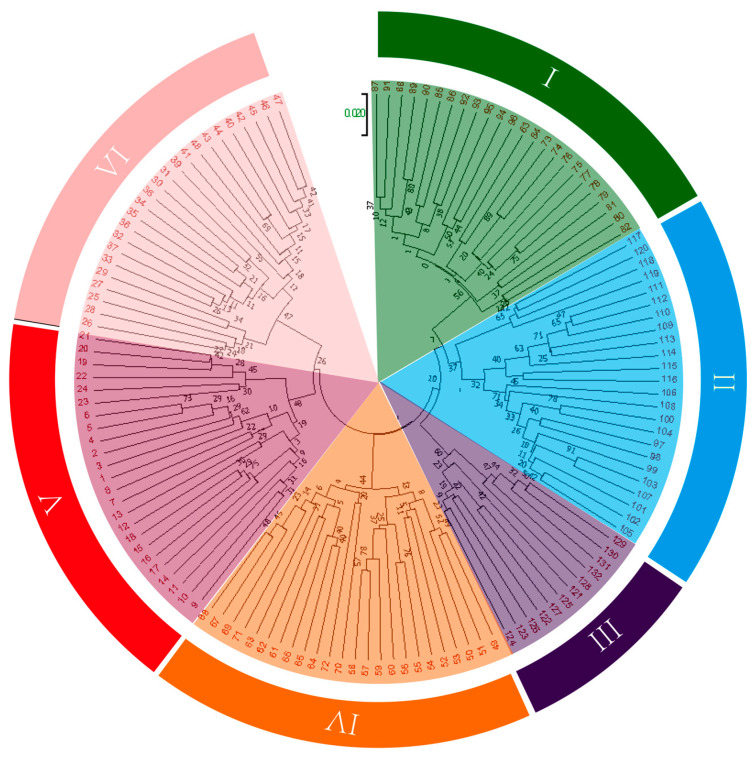
Clustering of 132 sugar beet varieties based on the three markers using UPGMA. The test materials were divided into six categories, distinguished by green, blue, purple, orange, red, and pink colors.

Based on the genetic distance matrix of the 132 test samples, clustering analysis was performed using the UPGMA method, and a dendrogram of the 132 sugar beet varieties in terms of relatedness was constructed (Figure 3). Most sugar beet varieties from the same breeding company were clustered together, and the genetic distance between them was small (Figure 3). However, few sugar beet varieties from different companies were clustered together. It may be due to the narrow genetic base of sugar beet or the exchange of sugar beet resources between different companies while breeding sugar beet varieties. At a genetic distance of 0.255, the test materials could be divided into six categories, which corresponded to the six colors in the figure. Category I (green) included 24 sugar beet varieties, most of which were from STRUBE. Category II (blue) included 24 varieties, mostly from SES VanderHave and BETASEED. Category III (purple) included 12 varieties, all from Maribohilleshog ApS. Category IV (orange) included 24 varieties, mostly from KWS SAAT SE and a few from STRUBE and Lion Seeds Ltd. Category V (red) included 24 varieties, mostly from SES VanderHave. Category VI (pink) included 24 varieties, mostly from KWS SAAT SE.

### 2.4. Analysis of Molecular Variance

We conducted an investigation into the genetic diversity of 132 exotic sugar beet cultivars utilizing AMOVA (analysis of molecular variance). The results showed that the main genetic variation of exotic sugar beet varieties was 95% within the population, and only a small part of the genetic variation was 5% among the various populations. The genetic differentiation among these populations was minor, quantified at 0.057 with a *p*-value less than 0.001, which suggests a considerable degree of genetic fluidity. Furthermore, the gene flow, denoted as Nm, was calculated to be equivalent to 4.98 immigrants per generation, as detailed in Table 2.

## 3. Discussion

A pivotal element in achieving breakthroughs in sugar beet breeding is the cultivation and deployment of superior germplasm resources. In recent years, researchers have systematically evaluated and analyzed a large number of crop germplasm resources by using SSR, SNP, and InDel markers to analyze their genetic diversity and population structure so as to facilitate subsequent germplasm improvement and innovation. Significant advancements in the analysis of genetic diversity and population structure have been realized in numerous crops, including soybean [30], rice [31], maize [32], wheat [33], tobacco [34], and potato [35], transitioning from rudimentary phenotypic assessments to sophisticated molecular-level investigations.

The collection of beet varieties from different regions is essential for the analysis and utilization of available beet germplasm resources. In this study, the selected sugar beet varieties were composed of 132 materials from France, the United States, the United Kingdom, Denmark, and Germany. These sugar beet varieties were introduced by six different breeding companies, and they have a high market share of sugar beet varieties, so it is representative and convincing to study the genetic diversity and population structure of high-quality exotic sugar beet varieties.

SSR marker has a wide range of applications and is simple and convenient. However, the bands amplified by InDel molecular marker technology are more accurate and clearer. In addition, newly developed CEAP primers in recent years can amplify clear and highly polymorphic bands, and CEAP markers are widely distributed in plants to prevent errors brought on by specificity and complexity. Given the advantages of these three markers, we decided to combine them to improve the efficacy of this study and yield superior and reliable findings when examining the genetic diversity of sugar beet germplasm. The evaluation of the twenty-seven primers suggested that all primers have PIC values greater than 0.5, and they were considered “highly informative” in the current study (Table 1), following the classification of Botstein et al. [36].

In this study, the genetic distances among sugar beet varieties from six different breeding companies ranged from 0.065 to 0.287, with small variations in genetic distances and insignificant differences in genetic backgrounds. This could be due to the narrow genetic base of sugar beet or the exchange of sugar beet resources among different breeding companies, which in turn resulted in smaller genetic distances [37]. Moreover, the exotic sugar beet varieties had significant gene exchange, which suggests a close genetic affinity among them. Liu et al. [38] used Indel and SSR molecular markers to study the genetic diversity of 21 red beet varieties. Through cluster analysis, they found that red beet varieties were scarce, and most of them were close relatives and had small genetic distances. Tehseen et al. [39] evaluated the potential of publicly available germplasm for sugar beet improvement by genetic diversity analysis with SNPs (single-nucleotide polymorphisms). Covering the whole genome of sugar beet was conducted using 1936 publicly available germplasm lines in the United States. The results confirmed the narrow genetic base of sugar beet. These conclusions are consistent with the findings of this paper. This indicates that the problem of a narrow genetic basis between the parents for the allocation of hybrid combinations has not been fundamentally solved.

This study provided a theoretical foundation for germplasm innovation and variety selection. In the future, it is necessary to make use of wild beet resources and various mutagenesis methods, increase the efforts to innovate beet germplasm resources, and expand the genetic basis of beet so as to better adapt to the development of future beet breeding.

## 4. Materials and Methods

### 4.1. Plant Material

A total of 132 exotic sugar beet varieties from six sugar beet breeding companies were used in this study [34 from SES VanderHave (France), 22 from STRUBE (Germany), 32 from KWS SAAT SE (Germany), 28 from Maribohilleshög ApS (Denmark), 8 from Lion Seeds Ltd. (UK), and 8 from BETASEED (USA)]. The number and name of sugar beet varieties and name of the breeding company are given in Table 3.

### 4.2. DNA Extraction of Sugar Beet Varieties

The cetyltrimethylammonium bromide (CTAB) protocol was employed for the isolation of genomic DNA from sugar beet samples [40]. DNA extraction was performed upon the emergence of 2–3 pairs of true leaves. The concentration and purity of the isolated DNA were evaluated utilizing a NanoDrop 2000/2000c Ultra-Micro UV–Vis Spectrophotometer (manufactured by Thermo Fisher, based in Madison, WI, USA). The DNA stock solution was subsequently diluted to a concentration of 10 ng/μL to create a working solution. The remaining DNA aliquots were preserved at a temperature of −20 °C for future experimental applications.

### 4.3. Primer Information Used in the Experiment

Three primers with high levels of polymorphism were selected to amplify the DNA samples of sugar beet varieties, including 27 pairs (bands) of 4 pairs of SSR primers, 3 pairs of InDel primers, and 20 CEAP makers (Table 4). The SSR primers 27,906, 11,965, and 57,236 were selected from earlier studies [41,42,43], whereas the SSR primers 2305 and InDel primers were designed and provided by the Laboratory of Molecular Genetics, Heilongjiang University, using the whole genome sequence of sugar beet. All CEAP primers were designed by the Germplasm Resources Laboratory of Guangxi University using the whole genome sequence of mangoes. All the primers were synthesized by Shanghai Bioengineering Co., Ltd. (Shanghai, China).

### 4.4. PCR Amplification Reaction System and Procedure

The PCR amplification volume was 5 µL, consisting of 2.5 µL 2 × Taq PCR Master Mix (BioTeke Corporation, Wuxi, China), 0.4 µL primer, 1.1 µL distilled water (ddH_2_O), and 1 µL DNA sample of the beet variety. PCR was performed using the Veriti 96-well thermal cycler (Ther-moFisher Scientific™, Shanghai, China).

The touchdown program was used for InDel and CEAP primers: predenaturation at 94 °C for 3 min, 15 s at 94 °C, annealing at 65 °C for 15 s, two cycles of 65–56 °C for every one degree down to 56 °C, and extension at 72 °C for 30 s. This was followed by 20 cycles of 15 s at 94 °C, 15 s at 55 °C, 30 s at 72 °C, and final extension at 72 °C for 5 min. The PCR reaction procedure used for SSR primers was predenaturation at 94 °C for 3 min, followed by 32 cycles of denaturation at 95 °C for 15 s, annealing at 57 °C for 15 s, and extension at 72 °C for 30 s, and finally, extension at 72 °C for 5 min.

The PCR products of SSR primers and InDel primers were detected by 8% nondenatured polyacrylamide gel electrophoresis, which was run for 1.5 h at a constant 180 V. The gel was stained with the nontoxic G-Red nucleic acid dye (BioTeke Corporation, Wuxi, China) and was photographed using a gel imager.

The PCR products of CEAP primers were detected using 2% agarose gel electrophoresis. A 5 µL amount of it was loaded on 2% agarose gel containing Gold View fluorescent nucleic acid dye. The horizontal electrophoresis cell voltage was set at 130 V, and electrophoresis was performed for 30 min. The agarose gel was taken out, and the band type was observed under the gel imager and photographed.

As mentioned above, the images generated under two different electrophoretic treatments were saved and exported for subsequent manual observation and recording in the corresponding Excel forms.

### 4.5. Statistical Analysis of Data

The outcomes of molecular marker detection were ascertained via the 0/1 assignment method. Initially, the amplified bands were meticulously analyzed manually, with their types documented in a corresponding table. Subsequently, to generate a binary data matrix comprising 0 s and 1 s, a value of “1” was allocated in instances where bands were present at identical positions, whereas a “0” was designated when no bands were observed.PopGen1.32 [44] was used to calculate genetic diversity indicators, including Na, Ne, Ho, He, I, and Nei’s. PowerMarker 3.25 [45] was used to calculate the genetic diversity index and PIC among different populations [46]. Based on genetic variation information, markers with PIC >0.5 were considered high-information markers, those with 0.5 > PIC > 0.25 as informative markers, and those with PIC <0.25 as “noninformative markers” [36]. The Structure v.2.3.4 software [47] was used to analyze the population structure of sugar beet varieties, and the optimal number of subgroups was calculated. This model-based software uses a Bayesian clustering method [48]. Using the Markov Chain Monte Carlo (MCMC) technique, the posterior probabilities were computed. The MCMC chains were run using a model that allowed for admixture and correlated allele frequencies with a burning period of 100,000, followed by 100,000 iterations. For each K-value, 10 runs were performed with K ranging from 1 to 10 to obtain an accurate estimation of the number of populations. The optimal number of subpopulations of the population was later determined by the rate of change in the posteriori probability values (∆K) [48] using the web-based program STRUCTURE HARVESTER [49]. By combining the data of the three different kinds of primers, a clustered dendrogram [36] of the 132 sugar beet varieties was generated based on Nei’s genetic distance [47], using the unweighted pair–group method with arithmetic averaging (UPGMA) [50]. Molecular variance analysis (AMOVA) and gene flow estimation are also involved. The original genotype data of exotic sugar beet varieties were used to calculate the variation, differentiation, and significance test in GenAlEx version 6.501 software. Gene flow (Nm) was calculated based on the genetic differentiation coefficient (Fst) obtained from GenAlEx version 6.501 [51].

## 5. Conclusions

In this study, we analyzed the genetic diversity and population structure of 132 sugar beet varieties from six sugar beet breeding companies. Their DNA samples were labeled using 27 pairs (bands) of primers. The results indicated that the genetic distance among 132 sugar beet varieties, both within the same company and between different companies, is small, and a significant portion of these varieties exhibit gene exchange. Consequently, the findings from these 132 sugar beet varieties reveal populations characterized by a low level of genetic diversity. This underscores the ongoing challenge of addressing the narrow genetic base issue in sugar beet breeding.

This study provides a theoretical basis for the innovation of beet germplasm resources and the selection of varieties. In the future, it is necessary to use wild sugar beet resources combined with various mutagenesis breeding, gene editing breeding, and other advanced technologies to broaden the genetic basis of sugar beet and better adapt to the development needs of future sugar beet breeding.

## Figures and Tables

**Table 1:** Characterization of genetic diversity via amplification using 27 primer pairs (bands) in 132 sugar beet varieties.

Number	Primers	Na	Ne	I	Ho	He	Nei’s	PIC
1	27,906	10	8.252	2.206	0.985	0.882	0.879	0.869
2	2305	13	6.585	2.115	0.930	0.851	0.848	0.840
3	11,965	15	7.023	2.321	0.879	0.861	0.858	0.847
4	57,236	9	6.269	1.959	0.791	0.844	0.840	0.847
5	D17	17	9.126	2.420	0.814	0.894	0.890	0.886
6	D31	15	8.047	2.344	0.830	0.879	0.876	0.871
7	D32	19	9.300	2.451	0.651	0.896	0.893	0.892
8	TGAC9	8	4.811	1.738	0.898	0.795	0.792	0.781
9	TGAC10	11	7.640	2.186	0.931	0.873	0.869	0.860
10	TCAC26	17	10.579	2.549	0.906	0.909	0.906	0.907
11	TCAC27	10	7.077	2.096	0.605	0.864	0.859	0.801
12	TGAC28	15	11.035	2.515	0.954	0.913	0.909	0.906
13	ACGTG4	9	5.483	1.828	0.207	0.822	0.818	0.781
14	GATAA1	12	7.818	2.202	0.754	0.876	0.872	0.876
15	GATAA2	11	4.001	1.806	0.487	0.753	0.750	0.773
16	TGAC23	6	3.591	1.470	1.000	0.724	0.722	0.682
17	TGAC19	9	7.189	2.071	0.887	0.865	0.861	0.866
18	TGAC18	9	6.833	2.015	0.418	0.860	0.854	0.700
19	TGAC12	7	4.048	1.559	0.858	0.756	0.753	0.737
20	TGAC6	11	6.154	2.017	0.714	0.795	0.838	0.781
21	TGAC7	8	5.701	1.846	0.857	0.843	0.825	0.817
22	TGAC20	11	8.220	2.193	0.780	0.883	0.878	0.859
23	TGAC21	10	6.410	1.995	0.954	0.847	0.844	0.831
24	TGAC22	10	6.013	1.950	0.930	0.837	0.834	0.824
25	ACGTG1	11	5.473	1.948	0.930	0.821	0.817	0.808
26	ACGTG3	10	6.593	2.007	0.902	0.852	0.848	0.830
27	AAAG28	5	3.155	1.368	0.221	0.688	0.683	0.639
	Total	298	182.426	55.175	21.073	22.683	22.616	22.111
	Mean	11	6.757	2.044	0.780	0.840	0.838	0.819

Note: 1–4 are SSR primers; 5–7 are InDel primers; 8–27 are CEAP primers; Na: number of observed alleles; Ne: effective number of alleles; Relevant calculation formulas: I (Shannon information index) =−∑(Pi)(lnPi); Ho (observed heterozygosity) $=-\sum i{\left(Pi\right)}^{2}$; He (expected heterozygosity) $=-\sum i{\left(Pi\right)}^{2}{\left(1-Pi\right)}^{2}$; Nei’s (Nei’s diversity index) $=1-\left(\sum {Pi}^{2}\right)/N$, N is the number of loci of the population. PIC (polymorphism information content) $=1-\sum_{i=1}^{l}Pi^{2}-\sum_{i=1}^{l-1}\sum_{j=i+1}^{l}2{Pi}^{2}{Pj}^{2}$, where *Pi* and *Pj* are the population frequency of the *i*th and *j*th.

**Table 2:** Analysis of molecular variance (AMOVA) results for 6 sugar beet varieties populations.

Source	df	SS	MS	PV%	*p*	Est. Var	Fst	Nm
Among populations	5	61.699	12.340	5%	<0.001	0.215		
Within populations	258	910.956	3.739	95%	<0.001	3.739		
Total	263	972.655		100%		3.954	0.057 *	4.98

Note: Source: variation. df: degrees of freedom; SS: sum of squares; MS: mean squared; Est. var.: estimates of variance; PV%: percentage of variation; Fst: fixation index; Nm: gene flow (Nm) value. * *p* < 0.001; Nm = (1 − Fst)/4Fst.

**Table 3:** Sugar beet (*Beta vulgaris* L.) varieties (n = 132) used in this study.

Number	Variety Name	Breeding Company	Number	Variety Name	Breeding Company
1	KWS126	KWS SAAT SE	67	Ma1	Maribohilleshög ApS
2	KWS127	KWS SAAT SE	68	Ma2	Maribohilleshög ApS
3	KWS128	KWS SAAT SE	69	Ma3	Maribohilleshög ApS
4	KWS129	KWS SAAT SE	70	Ma4	Maribohilleshög ApS
5	KWS130	KWS SAAT SE	71	Ma7	Maribohilleshög ApS
6	KWS131	KWS SAAT SE	72	Ma8	Maribohilleshög ApS
7	KWS132	KWS SAAT SE	73	Ma9	Maribohilleshög ApS
8	KWS133	KWS SAAT SE	74	Ma10	Maribohilleshög ApS
9	KWS134	KWS SAAT SE	75	Ma11	Maribohilleshög ApS
10	KWS136	KWS SAAT SE	76	Ma12	Maribohilleshög ApS
11	KWS137	KWS SAAT SE	77	Ma14	Maribohilleshög ApS
12	KWS138	KWS SAAT SE	78	Ma15	Maribohilleshög ApS
13	KWS139	KWS SAAT SE	79	Ma16	Maribohilleshög ApS
14	KWS140	KWS SAAT SE	80	Ma17	Maribohilleshög ApS
15	KWS141	KWS SAAT SE	81	Ma18	Maribohilleshög ApS
16	KWS158	KWS SAAT SE	82	Ma19	Maribohilleshög ApS
17	KWS0023	KWS SAAT SE	83	Ma20	Maribohilleshög ApS
18	KWS1130	KWS SAAT SE	84	MA22	Maribohilleshög ApS
19	KWS1131	KWS SAAT SE	85	MA23	Maribohilleshög ApS
20	KWS2407	KWS SAAT SE	86	23MH1	Maribohilleshög ApS
21	KWS2408	KWS SAAT SE	87	23MH2	Maribohilleshög ApS
22	KWS3473	KWS SAAT SE	88	23MH3	Maribohilleshög ApS
23	KWS3504	KWS SAAT SE	89	23MH4	Maribohilleshög ApS
24	KWS3505	KWS SAAT SE	90	23MH6	Maribohilleshög ApS
25	KWS6637	KWS SAAT SE	91	23MH7	Maribohilleshög ApS
26	KWS6653	KWS SAAT SE	92	23MH8	Maribohilleshög ApS
27	KWS7748	KWS SAAT SE	93	23MH9	Maribohilleshög ApS
28	KWS7772	KWS SAAT SE	94	23MH10	Maribohilleshög ApS
29	KWS8805	KWS SAAT SE	95	ST12528	STRUBE
30	KWS9147	KWS SAAT SE	96	ST12655	STRUBE
31	KWS9898	KWS SAAT SE	97	ST12763	STRUBE
32	KWS9962	KWS SAAT SE	98	ST12764	STRUBE
33	SX1535	SES VanderHave	99	ST12816	STRUBE
34	SX1537	SES VanderHave	100	ST12817	STRUBE
35	SV2427	SES VanderHave	101	ST12846	STRUBE
36	SV2538	SES VanderHave	102	ST12908	STRUBE
37	SV2674	SES VanderHave	103	ST12909	STRUBE
38	SV2675	SES VanderHave	104	ST13103	STRUBE
39	SV2676	SES VanderHave	105	ST13112	STRUBE
40	SV2761	SES VanderHave	106	ST13237	STRUBE
41	SV2762	SES VanderHave	107	ST13527	STRUBE
42	SV2763	SES VanderHave	108	ST13528	STRUBE
43	MK4185	SES VanderHave	109	ST13529	STRUBE
44	MK4205	SES VanderHave	110	ST13790	STRUBE
45	MK4241	SES VanderHave	111	ST13832	STRUBE
46	MK4245	SES VanderHave	112	ST13903	STRUBE
47	MK4256	SES VanderHave	113	ST13915	STRUBE
48	MK4257	SES VanderHave	114	ST13943	STRUBE
49	SR23001	SES VanderHave	115	ST15216	STRUBE
50	SR230010	SES VanderHave	116	ST15217	STRUBE
51	SR230011	SES VanderHave	117	L2301	Lion Seeds Ltd.
52	SR230012	SES VanderHave	118	L2302	Lion Seeds Ltd.
53	SR230013	SES VanderHave	119	L2305	Lion Seeds Ltd.
54	SR230015	SES VanderHave	120	L2306	Lion Seeds Ltd.
55	SR230016	SES VanderHave	121	L2307	Lion Seeds Ltd.
56	SR230017	SES VanderHave	122	LN001	Lion Seeds Ltd.
57	SR230018	SES VanderHave	123	LN002	Lion Seeds Ltd.
58	SR230019	SES VanderHave	124	LN003	Lion Seeds Ltd.
59	SR23002	SES VanderHave	125	Bts1714	BETASEED
60	SR230020	SES VanderHave	126	Bts1715	BETASEED
61	SR23004	SES VanderHave	127	Bts1730	BETASEED
62	SR23005	SES VanderHave	128	Bts3880	BETASEED
63	SR23006	SES VanderHave	129	Bts5940	BETASEED
64	SR23007	SES VanderHave	130	Bts6870	BETASEED
65	SR23008	SES VanderHave	131	Bts6871	BETASEED
66	SR23009	SES VanderHave	132	Bts7715	BETASEED

**Table 4:** The primers used in the study.

Primer Type	Primer Name	Primer Sequences (5′-3′)	Annealing Temperature
SSR	27906	F GAGCAGCAAACATGATAAGA	57 °C
		R GAAAACAGTGAGTATGGGTCTA	
	2305	F TACTAAAACCCTACGAACTCCA	55 °C
		R TACACCTGTGATTGTCAGAAGA	
	11965	F TTGAGTATTTTCGTCGGC	57 °C
		R CATCTACATCAGTTTTCCCTTC	
	57236	F TTGGAGAGAGAAAAGAGAGAAG	57 °C
		R ATCCCTTGACAGTAGAACTCC	
InDel	D17	F GATGGGGGAGATCCCAAC	Touch down
		R GCTTGACCCAGTGCCATC	
	D31	F CGCAGAGTGGTGTGTTGG	Touch down
		R TGGAGAATGGGTGTGCTG	
	D32	F GGGGGAGAGCAGTGGGTA	Touch down
		R AGCAGAGGAGGTGTGTGTGA	
CEAP	TGAC9	GCAGCTGAGAGTTGACGA	Touch down
	TGAC10	GCAGCTGAGAGTTGACGT	Touch down
	TCAC26	GCAGCTGAGGTTGACCAG	Touch down
	TGAC27	GCAGCTGAGGTTGACCTC	Touch down
	TGAC28	GCAGCTGAGGTTGACCGA	Touch down
	ACGTG4	GCAGTCAGATCACGTGAC	Touch down
	GATAA1	GCAGCTGCGTGGATAAAT	Touch down
	GATAA2	GCAGCTCGCTGGATAAAG	Touch down
	TGAC23	GCAGCTGAGGTTGACGAC	Touch down
	TGAC19	GCAGCTGAGGTTGACTAG	Touch down
	TGAC18	GCAGCTGAGGTTGACACA	Touch down
	TGAC12	GCAGCTGAGAGTTGACGG	Touch down
	TGAC6	GCAGCTGAGAGTTGACTT	Touch down
	TGAC7	GCAGCTGAGAGTTGACTG	Touch down
	TGAC20	GCAGCTGAGGTTGACTCA	Touch down
	TGAC21	GCAGCTGAGGTTGACTGT	Touch down
	TGAC22	GCAGCTGAGGTTGACTTC	Touch down
	ACGTG1	GCAGTCAGATCACGTGAA	Touch down
	ACGTG3	GCAGTCAGATCACGTGAG	Touch down

## Data Availability

The original contributions presented in the study are included in the article/Supplementary Materials, further inquiries can be directed to the corresponding author/s.

## Supplementary Materials

The following supporting information can be downloaded at: https://www.mdpi.com/article/10.3390/plants13212954/s1, Table S1: Genetic distance results of 132 sugar beet (*Beta vulgaris* L.) varieties.
